# Anteceding factors predicting absenteeism and presenteeism in urban area in Malaysia

**DOI:** 10.1186/s12889-019-6860-8

**Published:** 2019-06-13

**Authors:** Lei Hum Wee, Lena Lay Ling Yeap, Caryn Mei Hsien Chan, Jyh Eiin Wong, Nor Aini Jamil, Yogarabindranath Swarna Nantha, Ching Sin Siau

**Affiliations:** 1 Faculty of Health Sciences, Universiti Kebangsaan Malaysia, Jalan Raja Muda Abdul Aziz, Kuala Lumpur, Malaysia; 2 Stats Consulting Sdn. Bhd, Ara Damansara, Petaling Jaya, Selangor Malaysia; 3 Primary Care Department, Tuanku Jaafar Hospital, The Ministry of Health Malaysia, Bukit Rasah, Seremban, Negeri Sembilan Malaysia; 4 Faculty of Social Sciences and Liberal Arts, UCSI University, Kuala Lumpur, Malaysia

**Keywords:** Absenteeism, Presenteeism, Employees, Lower income, Malaysia

## Abstract

**Background:**

Organization productivity is strongly linked to employees’ socioeconomic characteristics and health which is marked by absenteeism and presenteeism. This study aims to identify anteceding factors predicting employees’ absenteeism and presenteeism by income, physical and mental health.

**Methods:**

An online health survey was conducted between May to July 2017 among employees from 47 private companies located in urban Malaysia. A total of 5235 respondents completed the 20-min online employee health survey on a voluntary basis. Chi-Square or Fisher’s exact tests were used to determine association between income with demographic and categorical factors of absenteeism and presenteeism. Multivariate linear regression was used to identify factors predicting absenteeism and presenteeism.

**Results:**

More than one third of respondents’ monthly income were less than RM4,000 (35.4%), 29.6% between RM4,000-RM7,999 and 35.0% earned RM8,000 and above. The mean age was 33.8 years (sd ± 8.8) and 49.1% were married. A majority were degree holders (74.4%) and 43.6% were very concerned about their financial status. Mean years of working was 6.2 years (sd ± 6.9) with 68.9% satisfied with their job. More than half reported good general physical health (54.5%) (*p* = 0.065) and mental health (53.5%) (*p* = 0.019). The mean hours of sleep were 6.4 h (sd ± 1.1) with 63.2% reporting being unwell due to stress for the past 12 months. Mean work time missed due to ill-health (absenteeism) was 3.1% (sd ± 9.1), 2.8% (sd ± 9.1) and 1.8% (sd ± 6.5) among employees whose monthly income was less than RM4,000, RM4,000-RM7,999 and over RM8,000 respectively (*p* = 0.0066). Mean impairment while working due to ill-health (presenteeism) was 28.2% (sd ± 25.3), 24.9% (sd ± 25.5) and 20.3% (sd ± 22.9) among employees whose monthly income was less than RM4,000, RM4,000-RM7,999 and over RM8,000 respectively (*p* < 0.0001). Factors that predict both absenteeism and presenteeism were income, general physical health, sleep length and being unwell due to stress.

**Conclusions:**

A combination of socioeconomic, physical and mental health factors predicted absenteeism and presenteeism with different strengths. Having insufficient income may lead to second jobs or working more hours which may affect their sleep, subjecting them to stressful condition and poor physical health. These findings demand holistic interventions from organizations and the government.

## Background

There is an increasing recognition in developing nations that health and socioeconomic factors are critical in influencing workplace productivity [1]. Suboptimal productivity arises from absenteeism, or being away from scheduled work [2–6]. Apart from missing work, presenteeism, which is characterized by coming to work regardless of impaired physical or psychological health problems [7, 8], is a greater source of aggregate productivity loss compared to absenteeism [9–11]. Presenteeism might worsen medical conditions, lower the quality of working life and lead to perceived decreased work efficiency as a result of reduced productivity [12]. The presenteeism phenomenon is more prevalent and difficult to estimate than absenteeism in its impact on workplace productivity [1, 13].

Private organizations with the primary aim of maximizing profit have historically depended on austerity measures such as downsizing, restructuring and other cost-cutting measures, which incurs added physical and mental burden on existing employees [14]. However, extant literature indicated that these measures could paradoxically result in losses caused by increased absenteeism and presenteeism among employees [14, 15]. Absenteeism and presenteeism costs are estimated to exceed medical and pharmaceutical expenses incurred by disability/illness [16, 17]. In the United States, absenteeism results in a total of $118 billion in losses [18], while in the UK, the cost of absenteeism and presenteeism from mental ill health annually was £8.4 billion and £15.1 billion respectively [19]. In Malaysia, the cost of absenteeism and presenteeism equated to 4.5% of the GDP in 2015 [20]. This situation may provide the economic incentive to invest in human capital through workplace wellness programmemes to achieve a positive return of investment via increased productivity, retention of experienced employees and reduced hiring costs [21, 22]. For example, a longitudinal study indicated that through implementing a seven-year employee wellness programmeme, the private sector conglomerate was able to generate a return of $1.46 for every dollar invested [23].

Recent studies have investigated the relationship between absenteeism and presenteeism with a number of lifestyle and health-related risk factors. Employees with higher absenteeism and presenteeism reported suffering from worse general physical health [24–26]. This includes suffering from heart conditions or diseases [27–29], migraine [30], asthma [31, 32], kidney disease [33] and diabetes [34]. In terms of mental health, higher rates of absenteeism and presenteeism are associated with stress [35–37], depression [10, 38, 39] and burnout [40]. Meanwhile, lifestyle factors such as sleeping habit and disorders [41–45] and smoking [46] are similarly linked to increased absenteeism and presenteeism. Inversely, higher self-reported job satisfaction [47, 48] and organizational commitment towards employees’ well-being [13, 49] are associated with lower absenteeism and presenteeism.

To date, there is a relative scarcity of research concerning the influence of income level on absenteeism and presenteeism, especially in Asia. Studies in developed countries such as Sweden indicated that lower income status is associated with both higher absenteeism and presenteeism [50]. In the US, a population study revealed that low-income earners were more than thrice as likely to indicate past-week presenteeism compared to higher income groups, even after controlling for gender, age, job type, and number of children [51]. Portuguese nurses with lower income were found to exhibit more presenteeism [52], as was reported among Spanish workers [53]. In addition, lower income status has been linked to a number of risk factors leading to absence and decreased work productivity, such as a higher prevalence of physical and mental illnesses [54].

In Asia, the work culture is largely characterized by a trend of high productivity loss compared to Western countries in spite of the higher number of hours spent at work. While Malaysian employees worked for about 44 h a week, there was a reported loss of 66 days annually on average to absenteeism and presenteeism, compared to UK employees who worked 35 h a week but lost only 30 days [55]. Thus, higher rates of absenteeism and presenteeism implicates not only organizational financial loss, but also a lower personal and work-related quality of life among Asian employees, characterized by long working hours and ill-health. However, to our knowledge, there is as yet no large-scale prevalence study in Southeast Asia which studies the predictive values of socioeconomic and health-related factors toward absenteeism and presenteeism.

The objectives of this study are to examine the relationships between income, demographics, health, workplace characteristics and absenteeism/presenteeism among private sector employees in Malaysia. This study also aims to determine the socioeconomic and health-related factors predicting absenteeism and presenteeism in this population.

## Methods

### Study design and participants

The Malaysia’s Healthiest Workplace by AIA is conducted by AIA Bhd., a leading insurer company in Malaysia. This is a cross-sectional online questionnaire survey on Malaysian working adults with the aim of providing employers strategies to support their employees’ healthy living goals and to improve company productivity [56]. A total of 47 private corporate companies in Malaysia participated in the study between 18th of May to 18th of July 2017. The majority of the participating companies are from sectors such as Financial, IT and Computer Software, Healthcare, Hospitality, Advertising, Manufacturing, Food, Consultancy, Property and Telecommunications.

Email invitations were sent to the employer (one representative from Human Resource Department from each organization) and all eligible employees of interested organization to participate in the survey. The employer was given an indication of the minimum sample size with the range of 10–70% depending on the size of the company to achieve results that can be interpreted statistically. A link to the survey was sent along with information to each employee after the organization agreed to participate. Participating organizations received a comprehensive report (Organizational Health Report) while the employees who completed the survey received a Personal Health Report. A total of 5369 employees responded to the survey.

### Employee survey questionnaire

This study analyzed data obtained from the Employee Survey Questionnaire, which was developed by AIA with oversight from an advisory board comprising of experts in workplace wellness, public health and mental health [56]. The questionnaire covers the multiple dimensions of self-reported health and well-being relevant to the workplace. The following are the variables of interest used in the analyzes:

#### Socio-demographics

Information on age, gender, ethnicity, marital status, education, occupation, employment length (in years), income, and presence of financial concerns were collected. Income was categorized into three categories which best approximates the bottom 40% (B40), middle 40% (M40) and top 20% (T20) strata for household income classification in Malaysia [57].

#### Physical health

Participants rated their physical health from “Very Good” to “Very Poor”. Long-term physical and health problems were measured by indication from participants on whether they suffer from kidney disease, diabetes and migraines.

#### Mental health

Participants rated their mental health from “Very Good” to “Very Poor”. Participants indicated the level of being unwell due to job stress as either “Yes, definitely”, “Yes, to some extent” and “No”.

#### Sleep length

Participants indicated sleep duration every day in hours.

#### Organizational factors

Information on the regularity of working hours and job satisfaction were collected. Job satisfaction was rated as “Agree”, “Neither Agree nor Disagree” and “Disagree”. Opportunities for job promotion was rated between “Strongly Agree” to “Strongly Disagree”.

### The work productivity and impairment – general health (WPAI-GH) questionnaire

The WPAI-GH is a questionnaire developed by Reilly and colleagues [58] with the aim of measuring absenteeism, presenteeism, productivity loss at work and daily activity impairment. This questionnaire was used extensively in both clinical and non-clinical populations to measure work productivity and impairment [59–61]. Outcomes are expressed as impairment percentages, with higher numbers indicating greater impairment and less productivity. The WPAI-GH contains six questions with a recall period of the past 7 days, including hours missed due to health problems (Q2), hours actually worked (Q4) and the degree of health affected productivity while working (Q5). Absenteeism is defined as the percentage of time miss from work because of health problems and is calculated by the formula Q2/(Q2 + Q4) × 100%. Presenteeism is measured by the degree health problems which affect productivity while working during the past 7 days on a rating scale ranging from 0 to 10, with 0 indicating that health problems had no effect on work and 10 indicating that health problems completely prevented the employee from working. The outcome is expressed as a percentage score representing the impairment due to health reasons while working, with higher numbers indicating greater impairment and less productivity, and was calculated by the formula (Q5/10) × 100%.

### Statistical analyses

The association between employees’ sociodemographic, job related characteristics, health status and income group were analyzed using univariate analyzes. Continuous variables were analyzed using Anova/Kruskal Wallis and categorical variables were analyzed using Chi-square (Table 1). The differences of the outcomes (absenteeism and presenteeism) between the employees’ income group were analyzed using Anova/Kruskal Wallis (Table 2). Multiple linear regression was carried out using an enter method (at *p* ≤ 0.05) to assess the strength of various predictors of the outcomes (Table 3). All analyses were performed using STATA version 13 (STATA Corp., TX, USA).

**Table 1:** Employee characteristics by income group (*N = 5235*)

Employee Characteristics	< RM4000	RM4000 - RM7999	>RM8000	Overall
Age, years	1856 (35.4%)	1548 (29.6%)	1831 (35.0%)	5235 (100%)
Mean (SD)	27.9 (6.0)	34.7 (7.6)	39.0 (8.5)	33.8 (8.8)
Median (IQR)	26 (6)	33 (10)	38 (12)	32 (12)***
(Min, Max)	(18, 59)	(21, 85)	(19, 67)	(18, 85)
Age years, No. (%)				
Age 18 to 24	609 (32.8)	41 (2.6)	54 (.03)	704 (13.5)***
Age 25 to 34	1004 (54.1)	840 (54.3)	545 (29.8)	2389 (45.6)
Age 35 to 44	197 (10.6)	496 (32.0)	770 (42.0)	1463 (28.0)
Age 45 to 54	43 (2.3)	136 (8.8)	367 (20.0)	546 (10.4)
Age 55 to 64	3 (0.2)	33 (2.1)	93 (5.1)	129 (2.5)
Age 65 above	0 (0.0)	2 (0.1)	2 (0.1)	4 (0.0)
Gender, No. (%)				
Male	592 (31.9)	572 (37.0)	812 (44.4)	1976 (37.8) ***
Female	1264 (68.1)	976 (63.0)	1019 (56.7)	3259 (62.3)
Ethnicity, No. (%)				
Malay	896 (48.3)	530 (34.2)	409 (22.3)	1835 (35.1) ***
Chinese	551 (29.7)	733 (47.4)	1066 (58.2)	2350 (44.9)
Indian	341 (18.4)	249 (16.1)	300 (16.4)	890 (17.0)
Others	68 (3.7)	36 (2.3)	56 (3.1)	160 (3.1)
Marital Status, No. (%)				
Single	1237 (66.7)	631 (40.8)	504 (27.5)	2372 (45.3) ***
Married	557 (30.0)	838 (54.1)	1177 (64.3)	2572 (49.1)
Separated/Divorced/Widowed	29 (1.6)	50 (3.2)	53 (2.9)	132 (2.5)
Prefer not to say	33 (1.8)	29 (1.9)	97 (5.3)	159 (3.0)
Education, No. (%)				
Less than University	558 (30.1)	418 (27.0)	363 (19.8)	1339 (25.6) ***
University degree or higher	1298 (69.9)	1130 (73.0)	1468 (80.2)	3896 (74.4)
Occupation, No. (%)				
Manager	113 (7.2)	513 (33.1)	962 (52.5)	1608 (30.7) ***
Professional	554 (29.8)	515 (33.2)	527 (28.8)	1596 (30.5)
Technician or junior professional	402 (21.7)	232 (15)	87 (4.8)	721 (13.8)
Clerical support worker	427 (23.0)	133 (8.6)	56 (3.1)	616 (1.8)
Service worker	64 (3.5)	24 (1.6)	8 (0.4)	96 (1.8)
Sales worker	125 (6.7)	54 (3.5)	23 (1.3)	202 (3.9)
Skilled worker	4 (0.2)	2 (0.1)	1 (0.1)	7 (0.1)
Financial Concern, No. (%)				
Don’t know	147 (7.9)	75 (5)	167 (9)	389 (7.4)
Yes, a little	589 (31.7)	509 (32.9)	801 (43.7)	1899 (36.3) ***
Yes, a lot	806 (43.4)	722 (46.6)	752 (41.1)	2280 (43.5)
No	461 (25.9)	317 (20.5)	278 (15.2)	1056 (20.2)
Employment Length, years				
Number	1856	1548	1831	5235
Mean (SD)	3.5 (4.5)	7.2 (7.0)	8.0 (8.0)	6.2 (6.9)
Median (IQR)	2 (3)	5 (8)	5 (9)	4 (7) ***
(Min, Max)	(0, 34)	(0, 40)	(0, 40)	(0, 40)
Work Irregular Hours, No. (%)				
Yes	496 (26.7)	303 (19.6)	304 (16.6)	1103 (21.1)
Job Satisfaction, No. (%)				
Agree	1223 (65.9)	1076 (69.5)	1306 (71.3)	3605 (68.9)**
Neither agree nor disagree	310 (16.7)	243 (15.7)	286 (15.6)	839 (16.0)
Disagree	323 (17.4)	229 (14.8)	239 (13.1)	791 (15.1)
Physical Health, No. (%)				
Very good	254 (13.7)	191 (12.3)	203 (11.1)	648 (12.4)
Good	1010 (54.4)	840 (54.3)	1007 (55.0)	2857 (54.5)
Fair	523 (28.2)	457 (29.5)	568 (31.0)	1548 (29.6)
Poor	65 (3.5)	57 (3.7)	45 (2.5)	167 (3.2)
Very poor	4 (0.2)	3 (0.2)	8 (0.4)	15 (0.3)
Mental Health, No. (%)				
Very good	370 (19.9)	288 (18.6)	352 (19.2)	1010 (19.3)*
Good	941 (50.7)	838 (54.1)	1024 (55.9)	2803 (53.5)
Fair	444 (23.9)	353 (22.8)	395 (21.6)	1192 (22.8)
Poor	87 (4.7)	59 (3.8)	49 (2.7)	195 (3.7)
Very poor	14 (0.8)	10 (0.7)	11 (0.6)	35 (0.7)
Sleep length, hours				
Mean (SD)	6.5 (1.2)	6.4 (1.1)	6.4 (1.0)	6.4 (1.1)
Median (IQR)	6 (1)	6 (1)	6 (1)	6 (1) ***
(Min, Max)	(1, 12)	(3, 24)	(2, 15)	(1, 24)
Health Condition Kidney Disease, No. (%)				
Yes	4 (0.2)	12 (0.8)	12 (0.7)	28 (0.5)
Health Condition Diabetes, No. (%)				
Yes	23 (1.2)	37 (2.4)	46 (2.5)	106 (2.0)*
Health Condition Migraines, No. (%)				
Yes	243 (13.1)	186 (12.0)	233 (12.7)	662 (13.7)
Unwell Due to Work Stress, No. (%)				
Yes, definitely	291 (15.7)	233 (15.0)	216 (11.8)	740 (14.1) ***
Yes, to some extent	981 (52.8)	763 (49.3)	823 (44.9)	2567 (49.1)
No	584 (31.5)	552 (35.7)	792 (43.3)	1928 (37.8)

*P < 0.05. **P < 0.01. ***P < 0.001

**Table 2:** Mean outcome of absenteeism and presenteeism among employees by income group (*N* = 5235)

	< RM4000	RM4000 - RM7999	>RM8000	p-value
Absenteeism				
Number				0.0066^*^
Mean (SD)	3.1 (9.1)	2.8 (9.1)	1.8 (6.5)	
Median (IQR)	0 (0)	0 (0)	0 (0)	
(Min, Max)	(0, 100)	(0, 100)	(0, 60)	
Presenteeism				
Number				0.0001^*^
Mean (SD)	28.2 (25.3)	24.9 (25.5)	20.3 (22.9)	
Median (IQR)	20 (40)	20 (40)	10 (30)	
(Min, Max)	(0, 100)	(0, 100)	(0, 100)	
Total	1856	1548	1831	5235

^*^p-values were obtained using Kruskal-Wallis Test

**Table 3:** Factors predicting absenteeism and presenteeism among employees in Malaysia (*N = 5235*)

	Absenteeism			Presenteeism		
	Coef.	[95% Confidence Interval]	*P*-value	Coef.	[95% Confidence Interval]	*P*-value
Income						
< RM4000^c^	1.00	–	–	1.00	–	–
RM4000 - RM7999	−0.53	(−1.10, 0.04)	0.067	−0.80	(−2.42, 0.83)	0.337
> RM8000	−1.46	(−2.03, −0.88)	< 0.001	−3.10	(−4.83, −1.38)	< 0.001
Age, years^b^	–	–	–	−0.31	(−0.39, −0.22)	< 0.001
Marital Status^a^						
Single^c^	1.00	–	–	–	–	–
Married	0.70	(0.20, 1.20)	0.006	–	–	–
Separated/Divorced/Widowed	2.05	(0.58, 3.52)	0.006	–	–	–
Prefer not to say	−0.78	(−2.12, 0.57)	0.258	–	–	–
Education^a^						
Less than University^c^	1.00	–	–	–	–	–
University degree or higher	−0.84	(−1.37, − 0.31)	0.002	–	–	–
Job Satisfaction^b^						
Agree^c^	–	–	–	1.00	–	–
Neither agree nor disagree	–	–	–	1.50	(−0.26, 3.25)	0.094
Disagree	–	–	–	4.63	(2.75, 6.50)	< 0.001
Physical Health						
Very good^c^	1.00	–	–	1.00	–	–
Good	0.97	(0.26, 1.68)	0.007	3.87	(1.60, 6.14)	0.001
Fair	1.44	(0.67, 2.22)	< 0.001	8.17	(5.60, 10.74)	< 0.001
Poor	2.21	(0.78, 3.64)	0.002	16.59	(12.29, 20.88)	< 0.001
Very poor	9.00	(4.77, 13.23)	< 0.001	25.73	(13.80, 37.66)	< 0.001
Mental Health^b^						
Very good^c^	–	–	–	1.00	–	–
Good	–	–	–	2.98	(1.05, 4.91)	0.002
Fair	–	–	–	6.24	(3.84, 8.63)	< 0.001
Poor	–	–	–	12.43	(8.43, 16.42)	< 0.001
Very poor	–	–	–	17.03	(9.01, 25.05)	< 0.001
Sleep Length	0.32	(0.11, 0.52)	0.002	−0.91	(−1.48, −0.35)	0.002
Kidney Diseases^a^						
No^c^	1.00	–	–	–	–	–
Yes	7.28	(4.22, 10.34)	< 0.001	–	–	–
Diabetes^a^						
No^c^	1.00			–	–	–
Yes	1.69	(0.09, 3.29)	0.038	–	–	–
Migraine^a^						
No^c^	1.00			–	–	–
Yes	1.18	(0.50, 1.86)	0.001	–	–	–
Unwell Due To Stress At Work						
Yes, definitely^c^	1.00	–	–	1.00	–	–
Yes, to some extent	−1.53	(−2.21, −0.85)	< 0.001	−6.82	(−8.73, −4.91)	< 0.001
No	−1.67	(−2.39, −0.95)	< 0.001	− 14.06	(− 16.12, − 12.00)	< 0.001

^a^Factor that only predict the Absenteeism

^b^Factor that only predict the Presenteeism

^c^Reference group

## Results

A total of 5235 participants responded to the online questionnaire (mean age = 33.8 years; sd ± 8.8). More than one third of the respondents’ monthly income were less than RM4,000 (35.4%), 29.6% between RM4,000-RM7,999 and 35.0% earned RM8,000 and above. Among those with monthly income less than RM4000, the mean age was 27.9 years (sd ± 6.0); a majority were single (66.7%), had a degree or higher (69.9%), worked as professional (29.8%), clerical (23.0%), technician or junior professional (21.7%) and service worker (3.5%). Nearly half of the participants (43.4%) had a lot of concern about their financial status. Mean years of working was 3.5 years (sd ± 4.5) with about one third working irregular hours (26.7%) and 65.9% reporting being satisfied with their job. Majority reported very good/good general physical health (68.1%) and mental health (70.6%). The mean hours of sleep were 6.5 h (sd ± 1.2), and 68.5% reported being unwell due to stress in the past 12 months. (Refer Table 1).

Mean work time missed due to ill-health (absenteeism) was 3.1 (sd ± 9.1), 2.8 (sd ± 9.1) and 1.8 (sd ± 6.5) among employees who earned less than RM4,000, RM4,000-RM7,999 and over RM8,000 in a month respectively (*p* = 0.0066). Mean impairment while working due to ill-health (presenteeism) was 28.2 (sd ± 25.3), 24.9 (sd ± 25.5) and 20.3 (sd ± 22.9) among employees who earned less than RM4,000, RM4,000-RM7,999 and over RM8,000 in a month respectively (*p* < 0.0001). (Refer Table 2).

Factors that predicted both absenteeism and presenteeism were income, general physical health, sleep length and being unwell due to stress. The absenteeism percentage decreased 0.53 (*p* = 0.067) and 1.46 (*p* < 0.001) if those earned between RM4000–RM7999 and RM8,000 and above increased by 1 respectively. The presenteeism percentage decreased 0.80 (*p* = 0.337) and 3.10 (*p* < 0.001) if those earned between RM4000–RM7999 and RM8,000 and above increase by 1 respectively. In terms of income, lower income employees recorded higher percentages of absenteeism and presenteeism. The absenteeism percentage for physical health increased 0.97 (*p* = 0.007), 1.44 (*p* < 0.001), 2.21 (*p* = 0.002) and 9.00 (*p* < 0.001) for 1 unit of increase in good, fair, poor and very poor physical health respectively. Meanwhile, the presenteeism percentage increased 3.87 (*p* = 0.001), 8.17 (*p* < 0.001), 16.59 (p < 0.001) and 25.73 (p < 0.001) for 1 unit of increase in good, fair, poor and very poor physical health respectively. The absenteeism percentage increased 0.32 and the presenteeism percentage decreased 0.91 (p = 0.002) with an hour increased in the sleep length respectively. The absenteeism percentage decreased 1.53 (*p* < 0.001) and 1.67 (p < 0.001) for 1 unit of increase some extent of and not being unwell due to stress at work respectively. The presenteeism percentage decreased 6.82 (*p* < 0.001) and 14.06 (p < 0.001) for 1 unit of increase some extent of and not being unwell due to stress at work respectively. Having insufficient income may be leading to second jobs or working more hours which may affect their sleep, subjecting them to stressful condition and poor physical health. Being divorced/separated (*p* = 0.006), kidney disease (p < 0.001), diabetes (*p* = 0.038) and migraine (*p* = 0.001) predicted higher absenteeism, while employees with higher education (*p* = 0.002) reported lower absenteeism. Both lower job satisfaction (p < 0.001) and good (p = 0.002), fair (p < 0.001), poor (p < 0.001) and very poor mental health (p = 0.001), as compared to very good mental health, were predictors of presenteeism respectively. (refer Table 3).

## Discussion

This study provides evidence on the importance of employees’ socioeconomic and health status as determinants of work productivity. In this sample, those from the lower income group were generally younger, single, female, less experienced, reported higher stress levels and financial concerns, lower job satisfaction, poor mental health and worked irregular hours. Studies indicated that there is an interplay between these characteristics which are common determinants to higher risk factors for absenteeism and presenteeism [12, 62]. This study further revealed that there is a stronger predictive effect of being in the lower income group on presenteeism compared to absenteeism as they may experience higher pressure from employers and co-workers to perform, in addition to having less authority over taking sick leave [63]. Moreover, the lower income group would be greater impacted by financial loss from absenteeism due to job insecurity [64, 65].

Employees who self-reported worse general physical health were also more likely to exhibit absenteeism and presenteeism behaviours, and the effect was stronger on the latter. However, employees who were suffering from specific physical illnesses (kidney disease, diabetes and migraines) were more likely to report absenteeism. These findings may be due to the fact that certain physical conditions predispose towards absenteeism, while others are more likely to lead to presenteeism [66]. The fact that specific illnesses lead to absenteeism is robust and rather self-explanatory – individuals who are ill need to recuperate and take time off to seek medical treatment, such as patients with kidney failure and complications arising from diabetes [33, 34, 67, 68]. However, the findings that worse self-reported physical health has a greater effect on presenteeism compared to absenteeism needs further explanation. Past studies have revealed that self-reported general health is strongly linked to less severe somatic symptoms such as chest pain, musculoskeletal symptoms and urinary retention [69]. Perhaps these symptoms are less severe than the specific illnesses mentioned above, and therefore may impair work performance but not to the extent of incurring absenteeism. However, poor physical health should not be considered in isolation as a single factor affecting absenteeism and presenteeism, as the interaction between individual and organizational problems need to be taken into consideration [21, 70, 71].

Apart from physical health, mental health was also found to influence absenteeism and presenteeism. Job stress level was found to predict both absenteeism and, to a greater extent, presenteeism. The effects of stress on worse physical and mental health outcomes, which may lead to lower work productivity, is well-documented [72, 73]. In addition, those who reported suffering from higher levels of stress were more likely to show up for work in spite of being ill, consistent with the findings of Brborovic and colleagues [35]. The greater effect of stress on presenteeism may be explained by the fact that conscientious employees who continue to work in spite of being ill were more likely to experience job stress due to their high performance standard [74] and the use of denial as a coping mechanism for job stress [75]. Hansen and Anderson [62] postulated that stress could exert compounded pressure for employees to exhibit presenteeism, and thus serve as a double risk factor which in turn exacerbates poor physical health and future absenteeism. In addition, the effect of stress is stronger on presenteeism compared to absenteeism, as employees who experienced more negative organizational environment such as heavier responsibilities, conflicts at work, perceived loss of control and lack of support reported exhibiting higher presenteeism [76, 77].

On the other hand, employees who self-reported worse general mental health also reported worse presenteeism. According to Evans-Lacko and Knapp [10], cultural contexts in which mental illness is stigmatized may prevent an employee from disclosing their mental illness status and take sick-leave due to mental health-related reasons. This is the case in Malaysia, where even though as high as one thirds of Malaysians were reported as suffering from a mental illness [78], there is still a lack of knowledge and a prevalence of stigma against individuals who are mentally ill or suicidal [79–81].

In terms of lifestyle factors, this study indicated that more hours of sleep predicted higher absenteeism. The findings on the positive relationship between sleeping hours and absenteeism are not consistent with previous studies where lesser and disturbed sleep were linked to higher rates of absenteeism [41–45]. Perhaps employees who slept more had underlying physical issues which led to absenteeism. Systematic reviews had revealed a U-shaped relationship between sleeping hours and overall health, in which the optimal hours of sleep were 7 to 8 h [82, 83]. Burton and colleagues [84] found that employees who slept more than 9 h were 2.39 times more likely to use medication to help with relaxation. Meanwhile, Strand’s [85] study indicated that mortality by heart disease was the strongest among those who slept more than 8 h a night in Taiwan. These findings point to probable underlying physical or mental health issues among employees who slept more. On the other hand, decreased sleep predicted higher presenteeism in this study. As was shown by Gingerich and colleagues [42], those who slept less may have decreased productivity at work due to heightened fatigue.

An organizational factor associated with higher presenteeism was lower job satisfaction. Past studies have generally focused on the impact of presenteeism on job satisfaction, citing job engagement and job addiction as reasons for employees to be present for work even though they are sick, which in turn led to lower job satisfaction [47]. Conversely, employees who experienced difficulty detaching from their work to the point of work addiction or workaholism were found to report lower job satisfaction [86, 87]. These are typically the employees who show up for work even though they are unwell [88].

In terms of sociodemographic factors, employees with higher education levels reported less absenteeism. Johansen and colleagues’ [65] research revealed a perception among highly educated employees that they are irreplaceable at work, leading to a reluctance to take sick leave. In addition, individuals with higher levels of education were reported to be healthier than those who received less education, both mentally and physically [89, 90], and therefore could be less prone to taking sick leave. Similarly, individuals who are divorced/separated reported the highest levels of absenteeism compared to married and single individuals. The necessity to attend divorce proceedings, the roll-over emotional effects of the divorce/separation and the ensuing lack of social support may be associated with higher absenteeism [91]. On the other hand, married employees with young children may experience heavier caretaking burdens and emotional exhaustion from juggling work-life responsibilities, compared to single employees, which in turn may also result in absenteeism [92–94].

This study has implications on the need for government and industry stakeholders to improve workplace productivity. The findings indicate an urgent need to target lower income group employees (those with monthly income less than RM4000), specifically to alleviate the occupational and environmental health issues which surround them. For example, at the macro level, health policy implementations need to strengthen its outreach to lower income groups, especially in removing barriers to mental and behavioral health treatments [95]. In terms of health promotion programmes, organizations need to create awareness on balancing physical and mental health, as well as employee engagement.

### Limitations and strengths

This study has a few limitations. Due to the cross-sectional design of this study, it is not possible to make causal inferences on the reported factors associated with absenteeism and presenteeism. Presenteeism could be best measured with a longitudinal study design using electronic daily diaries to capture prospective data [96]. This study employs single-item questions to measure absenteeism and presenteeism. In addition, data on employees’ physical health, mental health, sleep duration and job satisfaction were based on employees’ subjective self-report, which may be incur the risk of mnemonic bias. In order to prevent recall bias and increase accuracy, it is suggested that future studies should confirm sickness absence history with the employers’ record. In addition, the survey was disseminated through the human resources personnel of respective organizations. This could lead to social desirability in answering the questions. However, employees’ anonymity was assured at the outset of the study through the removal of identifiers in the online survey. We are not able to ascertain the participation rate for this study because we did not determine the number of employees who received the email blast in each organization. Moreover, we were unable to determine the influence of company sector on absenteeism and presenteeism as this information was not captured by the survey. We were also unable to identify factors within the organizations (e.g., organizational climate, leadership style, type and nature of business) which could be potential factors influencing absenteeism and presenteeism. Another limitation is that the findings may only be relevant for employees working in urban settings (i.e., corporate organizations) and does not include employees in rural areas. Hence, these predictors warrant further investigation in future studies. Finally, caution needs to be exercised in the interpretation of the study results as only private organizations took part in this study, and is therefore not generalizable to other sectors such as the public sector.

The strength of this study lies in the use of a large diverse sample in the exploration of the relative influence of selected socioeconomic and health-related factors on work attendance behaviors among employees in Malaysia. Being the first in the region, this study represents an important contribution to the literature. In addition, using the online survey method is able to efficiently capture data from a larger and diverse population for better generalisation of findings. Future studies should focus on the influence of workplace culture such as supervisory support, leadership style and employees’ rights for comparison with the West.

## Conclusions

A combination of socioeconomic, physical and mental health factors were found to predict absenteeism and presenteeism at different strengths among Malaysian employees in the private sector. These factors are specifically prominent in lower income employees which demands holistic interventions from organizations and the enhancement of government policies. Ultimately, strategies to reduce absenteeism and presenteeism such as health promotion programmemes should not only be implemented as a strategy to maximise the profit margin of organizations, but warrant our attention as a social justice issue contributing to the betterment of Malaysian working adults’ wellness and quality of life.

## Abbreviations

B40: Bottom 40%
GDP: Gross domestic product
M40: Middle 40%
RM: Ringgit Malaysia
SD: Standard deviation
T20: Top 205
WPAI-GH: The Work Productivity and Impairment – General Health questionnaire
